# First record of Cicadellidae (Insecta, Hemiptera, Auchenorrhyncha) from Eocene Sakhalinian amber

**DOI:** 10.3897/zookeys.886.38828

**Published:** 2019-11-05

**Authors:** Christopher H. Dietrich, Evgeny E. Perkovsky

**Affiliations:** 1 Illinois Natural History Survey, Prairie Research Institute, University of Illinois, 1816 S. Oak St., Champaign, IL 61820, USA University of Illinois Champaign United States of America; 2 Schmalhausen Institute of Zoology, National Academy of Sciences of Ukraine, B. Khmelnitskogo 15, Kiev, 01601, Ukraine Schmalhausen Institute of Zoology, National Academy of Sciences of Ukraine Kiev Ukraine; 3 Borissiak Paleontological Institute of the Russian Academy of Sciences, Profsoyuznaya Str. 123, Moscow, 117997, Russia Borissiak Paleontological Institute, Russian Academy of Sciences Moscow Russia

**Keywords:** amber insects, Bathysmatophorinae, leafhoppers, morphology, systematic paleontology, Xestocephalini

## Abstract

*Sakhalotettix eocenicus***gen. & sp. nov.**, the first leafhopper reported from middle Eocene Sakhalinian amber, is described and illustrated. The fossil cicadellid resembles modern Xestocephalini and Bathysmatophorini in some respects but, because of its unique combination of traits, cannot be placed with certainty in either group, or in any other modern cicadellid subfamily. It is, therefore, considered to be *incertae sedis* within Cicadellidae.

## Introduction

Leafhoppers (Cicadellidae) are one of the 10 largest families of insects and are presently among the most abundant herbivores, occurring in nearly every habitat that supports vascular plants. Although the oldest representatives of the family are recorded from the Lower Cretaceous (Hamilton 1990, 1992), the fossil record of this group remains sparse and poorly documented with fewer than 100 species formally described, most based on poorly preserved compression fossils (reviewed by Dietrich and Thomas 2018). Fossil cicadellids preserved in amber are known from the Cretaceous (Poinar and Brown 2017), Eocene (Szwedo 2002, 2005, Dietrich and Gonçalves 2014, Dietrich and Thomas 2018) and Miocene (Dietrich and Vega 1995). Many of these fossils have been assigned to modern subfamilies and have been used recently to calibrate molecular time trees (Dietrich et al. 2017, Johnson et al. 2018). The fossil leafhopper described below is important because it is the first representative of the family from middle Eocene Sakhalinian amber and exhibits a combination of morphological characters not yet reported among fossil or modern Cicadellidae. It may therefore contribute to knowledge of the evolution of major lineages of this family.

Numerous amber insects were collected in the south of Sakhalin Island, Russian Far East, by an expedition of the Paleontological Institute of Academy of Science of USSR in 1972 (Zherikhin 1978). Amber occurs on a beach near the village of Starodubskoye, Dolinsk District near the Naiba River mouth, Okhotsk Sea. The same fossil resin was found nearby at the Naiba River embedded in the coal of the Naibuchi Formation (Zherikhin 1978, Kodrul 1999).

The age of Sakhalinian amber has remained controversial for a long time (Kazantsev and Perkovsky 2019, and references therein). T.M. Kodrul (1999) convincingly demonstrated using geological and paleobotanical data a middle Eocene age of the Naibuchi Formation, in which Sakhalinian amber was found in situ (see also Baranov et al. 2015, Marusik et al. 2018). Rarity of ants and composition of mosses found in Sakhalinian amber confirm swampy environments in the Sakhalinian amber forest (Ignatov and Perkovsky 2013, Radchenko and Perkovsky 2016).

Sakhalinian amber belongs to the rumanite-type. Common for such fossil resins is a high degree of polymerization of the resin itself and deformation caused by thermal metamorphism during diagenesis. Insect inclusions in rumanite-type (particularly Sakhalinian) amber are therefore often deformed and have their internal cavity filled with resin (Rasnitsyn and Quicke 2002). This makes the inclusions more difficult to observe, compared to those European Baltic and Rovno succinite-type ambers. Sakhalinian amber is characterized by the small size of the pieces and supposedly rapid loss of viscosity (Kazantsev and Perkovsky 2019).

So far, about 1250 amber inclusions of insects and arachnids have been recorded from Sakhalinian amber, with aphids and chironomids most prevalent (Batelka et al. 2019, and references therein). Very unusual for amber faunas is the rarity of adult beetles in comparison with beetle larvae (Kazantsev and Perkovsky 2019).

Leafhoppers and planthoppers compose 23% of all hemipterans in a representative collection of late Eocene Rovno amber (Perkovsky et al. 2007) and 10% of all hemipterans in a representative collection of Eocene Baltic amber (Sontag 2003), but less than 0.3% of all hemipterans in middle Eocene Sakhalinian amber, possibly due to the small size of the pieces and low viscosity of the resin (our data).

Small typhlocybine nymphs jump less actively than other Auchenorrhyncha living in open habitats, and instead move laterally when danger threatens (Olmi et al., submitted). They are common in Rovno and Baltic ambers, but absent in Sakhalinian amber. So, apparently, they were very uncommon in the Sakhalinian amber forest. The new leafhopper described below is the only Auchenorrhyncha specimen known from Sakhalinian amber and only the second hemipteran species described from this amber (Shcherbakov 2007).

## Material and methods

The amber specimen was photographed using a Leica M165C microscope with Leica DFC 420 camera. Morphological terminology follows Dietrich (2005). Studied material is housed in the Paleontological Institute of the Russian Academy of Sciences, Moscow (**PIN**).

## Systematic Paleontology

### Order Hemiptera Linnaeus, 1758

#### Suborder Auchenorrhyncha Duméril, 1806

**Infraorder Cicadomorpha Evans, 1946**

**Superfamily Membracoidea Rafinesque, 1815**

**Family Cicadellidae Latreille, 1825, *incertae sedis***

##### Sakhalotettix gen. nov.

Taxon classificationAnimaliaHemipteraCicadellidae

32A3852A-E2FA-5E45-B1CC-E9C99BDFE67B

http://zoobank.org/7B8DD598-9497-476D-8D7C-8A18441CCDD9

###### Type species.

*Sakhalotettix eocenicus* sp. nov.

###### Diagnosis.

This genus differs from other known leafhoppers in having the following combination of traits: head with ocelli on crown near anterior margin distant from eyes; lateral frontal sutures well developed ventromesad of ocelli; frontoclypeus moderately convex, separated from eye by nearly half its width; gena emarginate below eye, exposing flaplike proepisternum; front femur row AV (anteroventral) with several short setae; female pregenital sternite nearly as long as all preceding sternites combined and acutely emarginate posteriorly.

###### Description.

Small, moderately slender. Head broad, crown moderately produced medially, texture uniformly shagreen, coronal suture extended nearly to anterior margin; anterior margin rounded in dorsal view, transition from crown to face broadly rounded, without transverse carinae. Ocelli well developed, on crown near anterior margin, approximately equidistant between eyes and midline, separated from lateral frontal suture by approximately one ocellar diameter, approximately even with anterior margins of eyes. Lateral frontal suture well developed, extended dorsad from antennal ledge then arched below ocelli and becoming obsolete near midline; temporal suture extended laterad of ocellus. Face broad, strongly convex, lower half closer to horizontal than vertical. Frontoclypeus irregularly rugose with distinct transverse muscle impressions, separated from mesal eye margin by approximately half frontoclypeal width; antennal ledges carinate but not strongly produced; antennal base well separated from eye, antenna shorter than half head width. Gena concavely emarginate below eye, exposing flaplike proepisternum. Lorum narrow, well separated from genal margin. Anteclypeus convex, tapered distally, extended beyond normal curve of gena. Rostrum extended slightly beyond front tronchanters.

Pronotum approximately as wide as head, moderately convex, finely punctate with numerous transverse striations, anterior margin slightly produced, lateral margin carinate, slightly shorter than eye, posterior margin slightly emarginate.

Front femur moderately broad basally, abruptly narrowed in distal two-thirds, seta AM1 enlarged and situated near ventroapical margin, row AV with numerous short setae extended over most of length, PV with several long, fine setae; tibia cylindrical, dorsal rows with numerous short setae and few widely spaced longer setae approximately as long as tibial width, row AV with approximately 14 macrosetae becoming progressively longer from base to apex, PV with fewer setae, restricted to distal half; tarsus three-fourths length of tibia. Middle femur broader and slightly shorter than front femur, setae of femur and tibia inconspicuous. Hind femur nearly reaching lateral margin of pronotum in repose; tibia with all four longitudinal setal rows well differentiated.

Pregenital abdominal sternite (VII) nearly as long as all preceding sternites combined, posterior margin deeply and acutely emarginate with lateral lobes acute.

###### Etymology.

The genus name, a masculine noun, combines the name of the type locality with “*tettix*”, a common suffix used in cicadellid genus names.

##### Sakhalotettix eocenicus sp. nov.

Taxon classificationAnimaliaHemipteraCicadellidae

BDBD34EB-0C65-58B7-891B-69A782AD5A1C

http://zoobank.org/3BE52080-FD1E-4704-A98F-CC31E97AB4D0

Figs 1 , 2

###### Description.

Structural features as in genus description.

###### Measurements.

Body length 4.2 mm; head width 1.3 mm; front femur length 0.8 mm, tibia length 0.95 mm; middle femur length 0.65 mm.

###### Material examined.

***Holotype***, female (?) PIN 3387/1085, Starodubskoye, Sakhalinian amber. middle Eocene.

###### Etymology.

The species name, *eocenicus*, refers to the age of the fossil.

**Figure 1.**
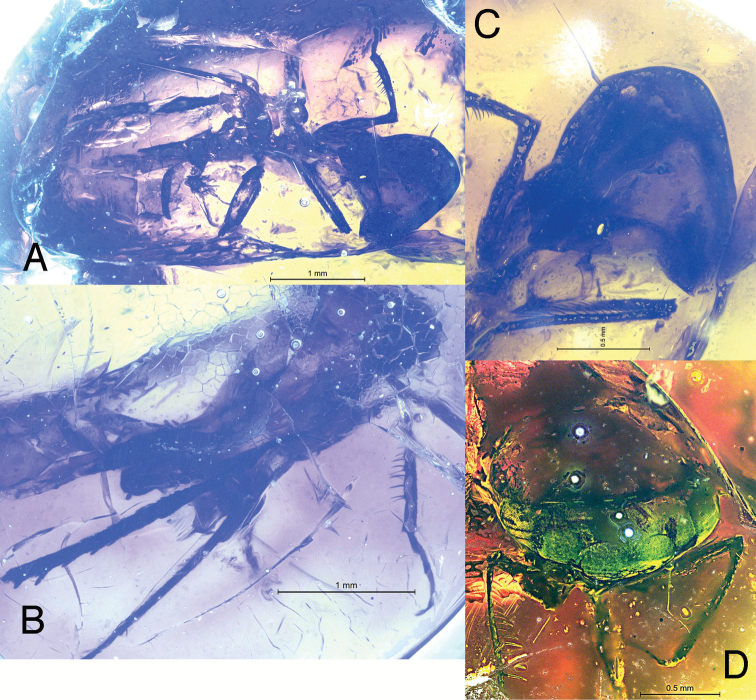
*Sakhalotettix eocenicus* gen. & sp. nov., holotype female **A** habitus, left ventrolateral view **B** habitus, right ventrolateral vie **C** detail of head and prothorax, left ventrolateral view **D** head and part of thorax, anterodorsal view.

**Figure 2.**
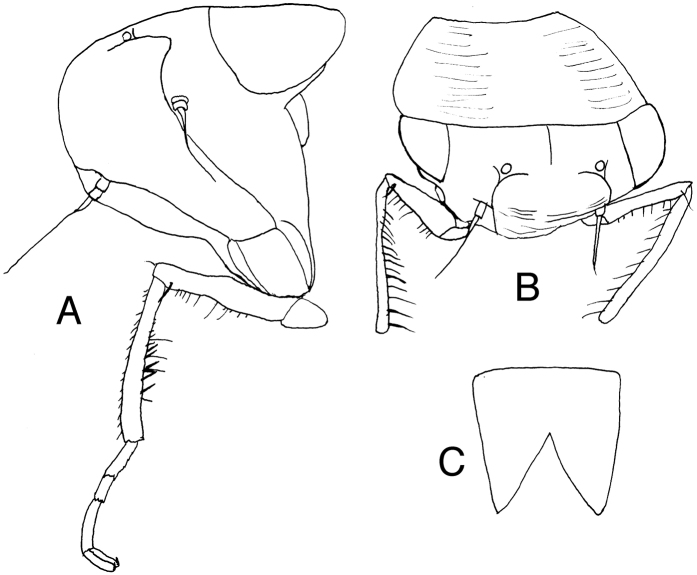
*Sakhalotettix eocenicus* gen. & sp. nov., holotype female **A** head, proepisternum and right front leg (part), left anterolateral view **B** head, pronotum and front legs, anterodorsal view **C** abdominal sternite VII, ventral view.

## Discussion

The fossil described above is moderately well preserved but several important structures, including the wings, hind legs, and abdominal terminalia are poorly visible. Nevertheless, the imperfect preservation of the specimen only partly accounts for the difficulty in placing *Sakhalotettix* within the current higher classification of Cicadellidae. In overall size and shape, the specimen resembles *Xestocephalus* Van Duzee (Aphrodinae, Xestocephalini), a modern cosmopolitan genus also recorded from lower-middle Miocene Dominican amber (Dietrich and Vega 1995). Two genera of Aphrodinae were previously described from the late Eocene (Baltic amber; Dietrich and Gonçalves 2014), but *Sakhalotettix* differs from these and modern aphrodines in having the gena concavely emarginate below the eye, exposing the proepisternum, and the distance between the eye and the frontoclypeus relatively broad. In the latter respects, *Sakhalotettix* resembles Bathysmatophorinae, a group represented by a few extant genera restricted to the Holarctic region but also recorded from late Eocene Baltic amber (Szwedo and Gebicki 1998, Szwedo 2005, Dietrich and Gonçalves 2014). However, in contrast to *Sakhalotettix*, all known Bathysmatophorinae are relatively large (macropterous adults > 5 mm total length) and have the ovipositor extended well beyond the pygofer apex. Unfortunately, the latter character is not visible in the holotype of *Sakhalotettix eocenicus* and the emarginate gena is shared with several modern subfamilies including Bathysmatophorinae, Cicadellinae, Ledrinae and Ulopinae.

*Sakhalotettix* is unusual in having the lateral frontal sutures well delimited ventromesad of the ocelli and extended nearly to the midline of the head. This presumably plesiomorphic trait occurs to various degrees in modern Cicadellinae, which differ in having the frontoclypeus strongly inflated and the ocelli usually situated well posterad of the anterior eye margins. Thus, based on the characters visible in the fossil, the new genus appears to have mixed affinities to at least three extant subfamilies (Aphrodinae, Bathysmatophorinae and Cicadellinae). Discovery of additional specimens with the wings and hind legs better preserved may help place the fossil with more certainty. For now, the unusual combination of plesiomorphic and apomorphic traits visible in the only specimen prevents us from placing it with confidence in any extant subfamily.

## Supplementary Material

XML Treatment for Sakhalotettix

XML Treatment for Sakhalotettix eocenicus

## References

[B1] Baranov V Andersen T Perkovsky EE (2015) Orthoclads from Eocene Amber from Sakhalin (Diptera: Chironomidae, Orthocladiinae). Insect Systematics & Evolution 46: 359–378. 10.1163/1876312X-45032122

[B2] Batelka J Perkovsky EE Prokop J (2019) Diversity of Eocene Ripiphoridae with descriptions of the first species of Pelecotominae and larva of Ripidiinae (Coleoptera). Zoological Journal of the Linnean Society. 10.1093/zoolinnean/zlz062 [in press]

[B3] Dietrich CH (2005) Keys to the families of Cicadomorpha and subfamilies and tribes of Cicadellidae (Hemiptera: Auchenorrhyncha). Florida Entomologist 88: 502–517. 10.1653/0015-4040(2005)88[502:KTTFOC]2.0.CO;2

[B4] Dietrich CH Allen JM Lemmon AR Moriarty Lemmon E Takiya DM Evangelista O Walden KKO Grady PGS Johnson KP (2017) Anchored hybrid enrichment-based phylogenomics of leafhoppers and treehoppers (Hemiptera: Cicadomorpha: Membracoidea). Insect Systematics and Diversity 1: 57–72. 10.1093/isd/ixx003

[B5] Dietrich CH Gonçalves AC (2014) New Baltic amber leafhoppers representing the oldest Aphrodinae and Megophthalminae (Hemiptera, Cicadellidae). European Journal of Taxonomy 74: 1–13. 10.5852/ejt.2014.74

[B6] Dietrich CH Thomas MJ (2018) New eurymeline leafhoppers (Hemiptera, Cicadellidae, Eurymelinae) from Eocene Baltic amber with notes on other fossil Cicadellidae. ZooKeys 726: 131–143. 10.3897/zookeys.726.21976 PMC5799744 29416387

[B7] Dietrich CH Vega FE (1995) Leafhoppers (Homoptera: Cicadellidae) from Dominican amber. Annals of the Entomological Society of America 88: 263–270. 10.1093/aesa/88.3.263

[B8] Duméril AMC (1806) XLIII Fam. Collirostres ou Auchénorinques. Zoologie analytique, ou méthode naturelle de classification des animaux, rendue plus facile à l’aide de tableaux synoptiques. Allais, Paris, 1–344. 10.5962/bhl.title.44835

[B9] Evans JW (1946) A natural classification of leafhoppers (Jassoidea, Homoptera). Part I. External morphology and systematic position. Transactions of the Entomological Society of London 96: 47–60. 10.1111/j.1365-2311.1946.tb00442.x

[B10] Hamilton KGA (1990) Homoptera. In: Grimaldi DA (Ed.) Insects from the Santana Formation. Lower Cretaceous of Brazil.Bulletin of the American Museum of Natural History 195: 82–122.

[B11] Hamilton KGA (1992) Lower Cretaceous Homoptera from the Koonwarra fossil bed in Australia, with a new superfamily and synopsis of Mesozoic Homoptera. Annals of the Entomological Society of America 85: 423–430. 10.1093/aesa/85.4.423

[B12] Ignatov MS Perkovsky EE (2013) Mosses from Sakhalinian amber (Russian Far East). Arctoa 22: 79–82. 10.15298/arctoa.22.11

[B13] Johnson KP Dietrich CH Friedrich F Beutel R Wipfler B Peters RS Allen J Petersen M Donath A Walden KKO Kozlov A Podsiadlowski L Mayer C Meusemann K Vasilikopoulos A Waterhouse RM Cameron S Weirauch C Swanson DR Percy D Hardy N Terry I Liu S Zhou LX Misof B Robertson HM Yoshizawa K (2018) Phylogenomics and evolution of hemipteroid insects. Proceedings of the National Academy of Sciences of the United States of America 115: 12775–12780. 10.1073/pnas.1815820115 30478043 PMC6294958

[B14] Kazantsev SV Perkovsky EE (2019) A new genus of soldier beetles (Insecta: Coleoptera: Cantharidae: Cantharinae) from Sakhalinian Amber. Paleontological Journal 53(3): 300–304. 10.1134/S0031030119030067

[B15] Kodrul TM (1999) Paleogene phytostratigraphy of the South Sakhalin. [Paleogenovaya paleostratigraphiya Yuzhnogo Sakhalina]. Transactions of GIN RAS 519: 1–150. [In Russian]

[B16] Latreille PA (1825) Homoptères. Homoptera. Seconde section. Familles Naturelles du Règne Animal, exposées succinctement et dans un ordre analytique, avec l’indiction de leurs genres. Baudouin Frères, Paris, 570 pp 10.5962/bhl.title.16094

[B17] Linnaeus C (1858) II. Hemiptera. Systema Naturae (10^th^ edn). Salvii, Holmiae, 824 pp.

[B18] Marusik YuM Perkovsky EE Eskov KYu (2018) First records of spiders (Arachnida: Aranei) from Sakhalinian amber with description of a new species of the genus *Orchestina* Simon, 1890. Far Eastern Entomologist. 367: 1–9. 10.25221/fee.367.1

[B19] Perkovsky EE Rasnitsyn AP Vlaskin AP Taraschuk MV (2007) A comparative analysis of the Baltic and Rovno amber arthropod faunas: representative samples. African Invertebrates 48(1): 229–245.

[B20] Poinar Jr G Brown A (2017) A new genus of leafhoppers (Hemiptera: Cicadellidae) in mid-Cretaceous Myanmar amber. Historical Biology 2017: 1–4. 10.1080/08912963.2017.1384472

[B21] Radchenko AG Perkovsky EE (2016) The ant *Aphaenogaster dlusskyana* sp. nov. (Hymenoptera, Formicidae) from the Sakhalin amber – the earliest described species of an extant genus of Myrmicinae. Paleontological Journal 50(9): 936–946. 10.1134/S0031030116090136

[B22] Rafinesque CS (1815) Analyse de la nature ou tableau de l’univers et des corps organisés. Jean Barravecchia, Palermo, 224 pp 10.5962/bhl.title.106607

[B23] Rasnitsyn AP Quicke DLJ (Eds) (2002) History of Insects. Kluwer Academic Publishers, Dordrecht, 517 pp 10.1007/0-306-47577-4

[B24] Shcherbakov DE (2007) Extinct four-winged precoccids and the ancestry of scale insects and aphids (Hemiptera). Russian Entomological Journal 16: 47–62.

[B25] Sontag E (2003) Animal inclusions in a sample of unselected Baltic amber. Acta Zoologica Cracoviensia 46: 431–440. [suppl. – Fossil Insects]

[B26] Szwedo J (2002) Amber and amber inclusions of planthoppers, leafhoppers and their relatives (Hemiptera, Archaeorrhyncha et Clypaeorrhyncha). In: Holzinger W (Ed.) Zikaden – Leafhoppers, Planthoppers and Cicadas (Insecta: Hemiptera: Auchenorrhyncha).Denisia 4: 37–56.

[B27] Szwedo J (2005) *Jantarivacanthus kotejai* gen. et sp. n. from Eocene Baltic amber, with notes on the Bathysmatophorini and related taxa (Hemiptera: Cicadomorpha: Cicadellidae). Polskie Pismo Entomologiczne 74: 251–276.

[B28] Szwedo J Gebicki C (1998) *Ambericarda skalskii* gen. et sp. n. from Baltic amber Homoptera: Cicadellidae). Polskie Pismo Entomologiczne 67: 179–184.

[B29] Zherikhin VV (1978) Development and replacement of Cretaceous and Cenozoic faunal assemblages. Trudy Paleontologicheskogo Instituta Akademii Nauk SSSR 165: 1–198. [In Russian]

